# Exploring the associations between physical activity, sleep, sedentary behaviour, and mental health in young German children

**DOI:** 10.1186/s44167-026-00104-3

**Published:** 2026-06-11

**Authors:** Katarina Osojnicki, Berit Brandes, Mirko Brandes, Hajo Zeeb, Lori Ann Vallis, Christoph Buck

**Affiliations:** 1 Department of Human Health Sciences, University of Guelph, Guelph, ON Canada; 2 Leibniz Institute for Prevention Research and Epidemiology— BIPS, Achterstr. 30, 28359 Bremen, Germany; 3 Faculty 11 Human and Health Sciences, University of Bremen, Bremen, Germany

**Keywords:** Children, Sleep, Physical activity, Sedentary behaviour, Mental health, Accelerometry

## Abstract

**Background:**

Early childhood is a period of rapid development; research shows that the formation of healthy habits during this period can result in higher physical fitness levels and better sleep, but also long-term improved mental health and wellbeing. Despite structures supporting physical activity (PA) and related behaviours, many German children under 6 years do not achieve the recommended levels of PA, sedentary behaviour (SED), and sleep; this in turn can hinder the formation healthy lifestyle habits in the early years, and also lead to long-term poor mental health, later in life. Thus, this study aimed to explore the associations between device-based measured PA with nighttime sleep, SED, and mental health in German children under six years of age.

**Methods:**

PA, sleep, and SED were assessed at baseline and 1-year follow-up using wrist-worn GENEActiv accelerometers sampled at 100 Hz. The R-package GGIR (version 3.1.1) was used to derive light-intensity PA (LPA), moderate-to-vigorous-intensity PA (MVPA), total PA (TPA), inactivity (proxy for SED), total night sleep time (TST), and sleep efficiency (SE). Parents answered on children’s mental health using the Strengths and Difficulties Questionnaire (SDQ). Linear mixed models, were used to estimate cross-sectional associations from repeated measures of PA intensities with sleep, SED, and mental health, adjusting for age and sex of the child, parental education, migration background, urbanity, and household income.

**Results:**

We investigated 212 children aged 2–6 years (51% female at baseline, 5.2% overweight or obese). At baseline, children spent on average 418.7 min/day inactive, 252.6 min/day in MVPA, and 480 min/day asleep, with a SE of 80%. The results indicated relevant associations of LPA, MVPA, or TPA with SED, SE, or TST, but no association of PA variables and SDQ.

**Conclusion:**

Given the rapid developmental changes in early childhood, it is essential to track 24-hour movement behaviors, and mental health over time using device-based measures to better inform strategies that promote lifelong physical and mental wellbeing. Future multi-component interventions should be explored to determine potential synergistic benefits for mental health.

## Introduction

The World Health Organization’s (WHO) messaging around their 2019 published guidelines on physical activity (PA), sedentary behaviour (SED), and sleep for children under five years is clear: for children to grow up healthy, they need to spend less time sitting in front of screens or being restrained, have more time for active play, and have better quality of sleep [1]. A recent study by Kuzik et al., [2] showed the importance of moderate to vigorous PA (MVPA) for physical development in early childhood. In contrast, they found that stationary time can have mixed findings on cognitive development outcomes, e.g. reading may be beneficial while screen time may be detrimental. We know from many years of research that PA is beneficial for children as it enhances psychosocial wellbeing, improves motor skill development, improves bone health, and contributes to maintaining a healthy weight [3]. Similarly, adequate sleep has been favourably associated with improving emotional regulation, increasing academic performance, enhancing wellbeing and lowering adiposity [4]. However, in recent years, there has been a notable rise in SED among young children, driven in part by increased access to screen-based activities, less desire for active playtime, and lifestyle shifts that do not prioritize movement [1].

Early childhood is a period of rapid development, thus a positive change in a child’s 24-hour activity patterns can result in not only higher physical fitness levels and better sleep, but also improved mental health and wellbeing [1]. In turn, research has shown that these behavioural modifications can help prevent childhood obesity and associated diseases later in life [4, 5]. Overall research has elucidated that PA and sleep levels in childhood have important clinical implications in addition to increased fitness and the development of key motor skills in children and youth [6]. It is important to note that while PA is generally linked to better sleep hygiene in adults, the directionality of the relationship between PA and sleep in early childhood (< 7 years) remains unclear [7]. Sleep patterns can be influenced by physiological, genetic, psychological, and environmental factors [8]. While some previous research suggests a bi-directional relationship between PA and sleep, various confounding factors influence these behaviours throughout life, particularly in early childhood, when children are highly dependent on their caregivers’ attitudes toward co-sleeping and prioritization on PA [9, 10]. Given the many factors shaping young children’s sleep and PA patterns, there is a clear need for further research to better understand the complexity of these relationships.

Despite published movement behaviour guidelines, initiatives, and structures supporting PA and related behaviours, many German children under 6 years do not achieve the recommended levels of PA, SED, and sleep. For example, self-reported data from 12,981 German children and adolescents aged 3 to 17 years revealed that only 26.0% achieved a minimum of 60 min of MVPA daily [11]. Research on PA, SED, and sleep in young children is often limited by the use of subjective measures, which are prone to e.g. recall bias and social desirability effects [12]. Furthermore, in a recent systematic review, Laurent et al., [7] identified thirty-six studies investigating the relationship between PA and sleep in young children. Of these, only three studies used device-based measures of both PA and sleep in children between two and less than seven years of age [13–15]. This highlights the need for more device-based methods to better understand the association between PA, SED, and sleep in young children [15]. Although assessments of 24-hour movement behaviors remain susceptible to bias, particularly due to decisions on intensity cut-points, and sleep algorithms that are less accurate outside the validated age-range [16, 17], this knowledge is critical for understanding how 24-hour movement behaviours jointly affect health outcomes beginning in early childhood onward.

Associations between mental health and PA involvement have been studied for decades and positive associations have been reported for both school-aged children and adolescents [18]. Numerous studies have exemplified that youth who engage in higher intensities of PA experience better physical and mental health and enhanced psychosocial wellbeing compared to their more sedentary counterparts [19–21]. To date, most of these published studies have focused on these associations in older children and adolescents (e.g. Padmapriya et al. [22], Rose and Soundy, [19], Saunders, [21], whereas there is a clear gap in the literature examining how devices-based measured PA may influence emotional/behavioural wellbeing in young children, specifically in those under six years of age.

Therefore, the aim of the current study was to explore whether PA was associated with SED, and nighttime sleep using device-based measures, and explore the association of PA and sleep with mental health in a sample of German preschool children. We hypothesized that children who engage in a 24-hour movement pattern that has higher PA intensities (i.e. more MVPA and total PA), less time in SED, and better sleep (i.e. higher sleep efficiency; more total hours of sleep), will demonstrate better outcomes related to mental health (i.e. less difficulties with emotional and behavioural regulation).

## Methods

### Study data

Data was collected at baseline between September and December 2016 and 1-year follow-up (FUp) data was collected between September and December 2017. During data collection, a subsample of children from the larger, Germany-wide cluster-controlled trial was selected to wear accelerometers to evaluate the impact of ‘*JolinchenKids – Fit and Healthy in Daycare*’ intervention in daycare facilities, created by a German health insurance company AOK. Full details on the inclusion criteria can be found in a previous publication [23]. The original study was registered at the German Clinical Trials Register (DRKS00011065) on 16th of September 2016. The current analysis has been conducted using the entire sample of children that provided valid accelerometer data at baseline and FUp, as no significant differences were observed in PA and SED following the PA intervention [24]. Parents provided written consent for their children to participate in the study, while children provided additional verbal agreement. The study obtained ethical approval by the Medical Association in Bremen (Protocol # HR/RE − 522).

### Anthropometric measurements

In brief, two study nurses, trained two researchers in advance, collected anthropometric data at both time points. Children wore their own comfortable clothes and height (to the nearest 1 cm) and body weight (to nearest 0.1 kg) was measured (Seca^®^ type 213 stadiometer; Invicta Plastics Ltd, Leicester, UK). Body mass index (BMI) was calculated as weight in kilograms divided by body height in meters squared. Children were classified as underweight/normal or overweight/obese, according to age- and sex-specific cut-offs according to by Cole and Lobstein [25].

### Accelerometer measurements

PA, sleep, and SED were measured using GENEActiv triaxial accelerometers, initialized according to manufacturer’s instructions, and with a sampling rate of 100 Hz (Activinsights Ltd, Kimbolton, UK). Participants wore the device taped to their left wrist using Tyvek tape for 7 consecutive days, 24 h per day, at both baseline and FUp [26]. To cross-validate accelerometer data, children’s parents were asked to fill out a logbook to record non-wear time. Non-wear logbooks in combination with accelerometer data have been shown to increase data accuracy by comparing actual movement time from perceived movement time [27]. Specific nights or days were excluded from sleep and/or PA analyses if the child’s parent documented non-wear time of the accelerometer at night and/or day, or if visual inspection of the GGIR output plots suggested the accelerometer was not worn at night and/or day [28].

Raw accelerometer data were processed using the GGIR package (version 3.1.1) in R (version 4.4.0). As recommended in the GGIR vignette, the Euclidean Norm Minus One with Adjustment (ENMOa) method was used considering the Roscoe et al., [29] cut-point to remove the effect of gravity from the raw data [30]. Negative values were converted to absolute values, ensuring the isolation of clean PA data without gravitational noise [30]. Recent research suggests minimal differences in sleep period detection between nights processed with and without sleep logs for children [31]. Therefore, since sleep log information was not collected for participants’ day and nighttime sleep, we were unable to account for any daytime naps taken by the children and had to rely on the van Hees sleep algorithm in the GGIR R statistical package [28, 32] for all sleep metric calculations.

The van Hees et al., [32] algorithm was used to detect sleep and periods of inactivity. In brief, this algorithm identifies sustained periods of inactivity (SIB) lasting five minutes or longer, during which the arm angle relative to the horizontal plane remains five degrees or less, classifying this as sleep [32]. SIB within the nocturnal sleep period time (SPT) window were considered sleep episodes, and SIB outside the SPT window were considered rest, potential naps, or missed short episodes of device non-wear time [28]. The Distribution of Change in Z-Angle (HDCZA) algorithm in GGIR determines the SPT window by identifying the start and end of the longest inactivity block within a 24-hour period [33].

To balance the trade-off between maintaining a sufficient sample size and including waking hours of pre-school children in the data processing [34], we considered days with a minimum of 12 h of wear time measured from midnight to midnight valid for the analyses, instead of 16 h which is set as the default in GGIR. Inclusion criteria required participants to obtain a minimum of four nights of valid sleep data (weekdays and/or weekends including one weekend-day; [35]) and three days of valid PA data (≥ 12 h/day) including at least one weekend-day after exclusion of non-weartime [34]. Participants who refused to wear the accelerometer and/or failed to meet wear time criteria were excluded from the study [16].

### 24-hour movement behavior variables

Time spent awake was separated into three categories: inactivity, which is used as a proxy for SED [36], light PA (LPA), and MVPA. PA variable intensity thresholds were based on Roscoe et al.,‘s [29] cut-points for preschoolers (ages 4–5 years), whereby LPA and MVPA were defined by accelerations of ≥ 61.8 *m*g and ≥ 100.4 *m*g, respectively, and inactivity was defined by accelerations < 61.8 *mg* and used as a proxy for SED. PA variables computed in GGIR and used in the analyses included LPA, MVPA, total PA (TPA; the sum of LPA and MVPA), in addition to measured time spent engaging in SED. The sleep variables computed in GGIR included total sleep time (TST; hours asleep in bed at night, based on the accumulated SIB within the SPT) and sleep efficiency (SE; percentage of time spent asleep versus time spent in bed intending to sleep at night). SE was manually calculated due to the absence of the sleep logs using the following equation:

1 $$SE = \left( {{{Sleep\;Duration\;In\;SPT} \mathord{\left/ {\vphantom {{Sleep\;Duration\;In\;SPT} {SPT\;Duration}}} \right. \kern-\nulldelimiterspace} {SPT\;Duration}}} \right)*100$$

Where *Sleep Duration in SPT* was defined as the actual nocturnal sleep time within the SPT and *SPT Duration* was defined as the total time window allocated for sleep at night (see, https://wadpac.github.io/GGIR/articles/GGIRoutput.html). TST was used to assess sleep quantity, while SE were used to evaluate sleep quality.

### Mental health assessment

Children’s’ mental health was assessed with the Strengths and Difficulties Questionnaire (SDQ). The SDQ is a screening tool that can be used to assess social, emotional, and behavioural difficulties in children aged 2 to 17 years [37]. It is frequently used in longitudinal and intervention-based research and its clinical cut-offs have been validated as a baseline for identifying mental health concerns [37, 38]. Parents completed the SDQ to assess their child’s mental health and data was scored based on the cut-off categorization for 4- to 17-year-olds (see [39]).

### Statistical analyses

Descriptive statistics (e.g. proportions) were calculated to summarize the children’s characteristics (e.g. sex, age; Table 1). Means and standard deviations were used to describe PA, SED, sleep, and mental health at baseline and FUp (Table 2). Linear mixed models (LMM) were used to investigate cross-sectional associations between PA variables, SED, sleep variables and mental health (SDQ) accounting for repeated measures using a random effect; the flexibility of mixed models permitted the use of unbalanced participant data at baseline without observations at FUp. Models were adjusted for age and sex of the child, household income, parental education (using definitions from [40]), and migration background (using definitions from [41]). For each PA outcome (LPA, MVPA, TPA), either sleep (TST and SE), SED, or mental health (SDQ) metrics was used as the response variable. Therefore, 12 separate models in total were performed. Significance level was set to α = 0.05, but analyses were not adjusted for multiple testing. Statistical analyses were conducted using SAS 9.4 (SAS Institute Inc, Cary, NC) using the GLIMMIX procedure to estimate LMM.

**Table 1:** Characteristics of study population at baseline and follow-up

Variable	Baseline	Follow-up
Children, *n*	215	155
*Child sex, %*		
Male Female	49.8 50.2	47.7 52.3
*Age category, %*		
2–4 years 5–6 years,	56.3 43.7	17.4 82.6
Body mass index*, %		
Normal Overweight/obese NA	92.6 4.7 2.8	92.3 5.8 1.9
*Monthly household income, %*		
< €2000 €2000 - €3000 > €3000 Missing	17.2 26.0 57.9 8.8	13.5 27.1 51.0 8.4
*Parent highest educational level, %*		
Low Medium High Missing	10.2 53.0 31.6 5.1	5.8 61.3 28.4 4.5
*Migration background, %*		
None One or two sided Missing	77.7 18.6 3.7	76.1 16.8 7.1
*Urbanity (districts)*		
Rural (Population < 20,000) Small or large cities (Population ≥ 20,000)	47.4 52.6	47.1 52.9

*For BMI classification see Cole [25]

**Table 2:** Descriptive values for average physical activity, sedentary behaviour, sleep and mental health variables at the two time points

Variable	Baseline			Follow-Up		
	*N*	Mean	StD	*N*	Mean	StD
SED (mins/d)	212	418.7	85.0	153	408.0	103.6
MVPA (mins/d)	212	252.6	64.3	153	264.5	75.5
LPA (mins/d)	212	112.7	15.9	153	110.3	19.4
TPA (mins/d)	212	365.4	63.8	153	374.8	72.4
TST (hours/d)	186	8.0	0.7	135	8.0	0.6
SE (%)	186	80	0.0	135	80	0.0
SDQ Score	213	8.0	4.9	153	7.0	4.2

StD, standard deviation; SED, sedentary behaviour; MVPA, moderate-vigorous PA; LPA , light physical activity (PA); TPA, total PA; SE, sleep efficiency; TST, total sleep time; SDQ, Strengths and Difficulties Questionnaire. Note. See Goodman [39] for SDQ scoring cut-off categorizations

## Results

**Fig. 1:**
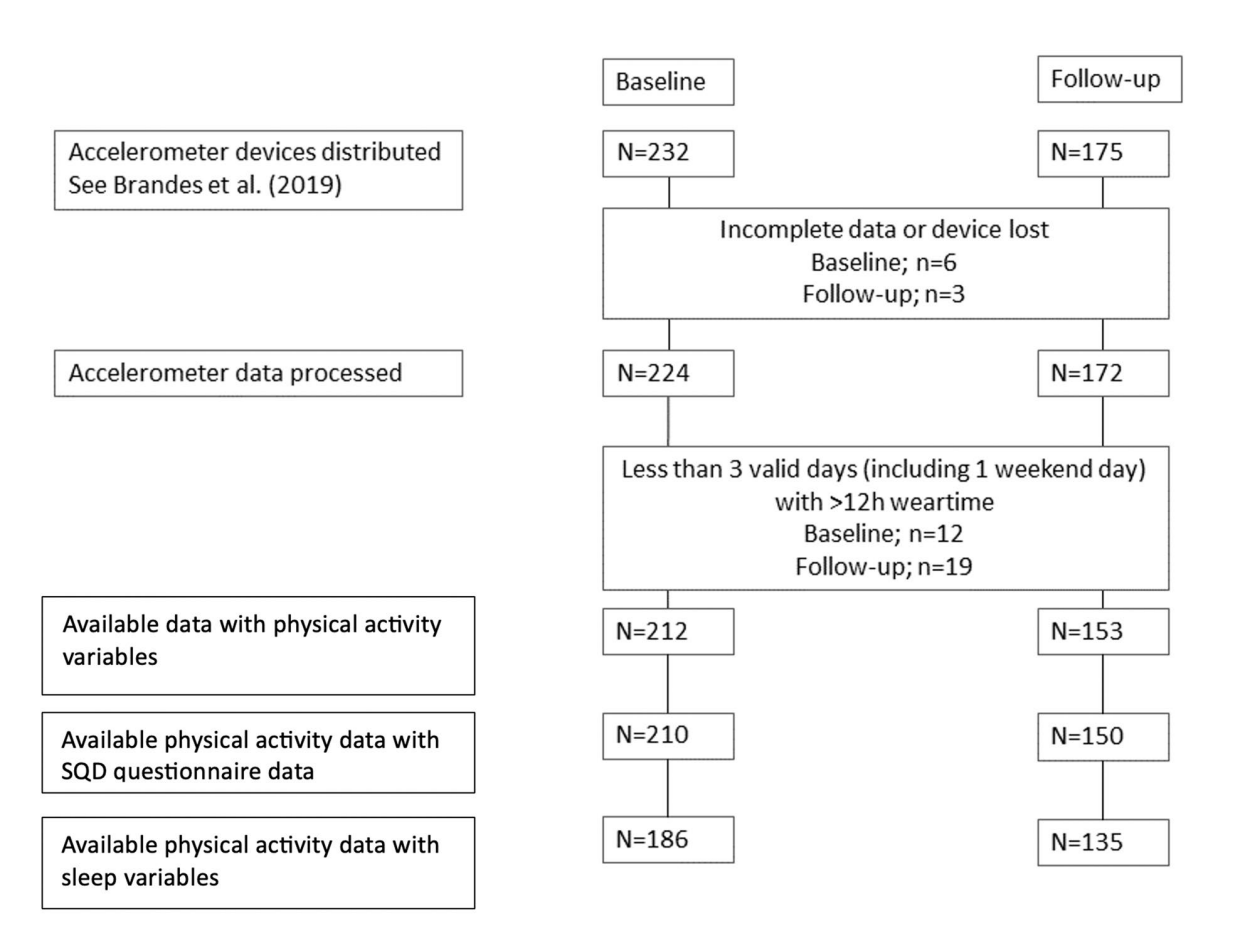
Data Flowchart describing exclusion criteria and sample size

Accelerometers were provided to 232 children at baseline and 175 children at the 1-year follow-up at 23 daycare facilities across Germany. A total of 212 children between the ages of 2 to less than 7 years were included in the final analysis of this study. Figure 1 summarizes the exclusion of participants per wave with regard to insufficient weartime. Please see Brandes et al., [24] for details regarding sample size and retention.

### Demographics of children

Demographic characteristics of our subsample at baseline and follow-up are reported in Table 1. A total of 50% of our participants came from families with a monthly household income of over €3000, 51% had parents with a medium education level, and 76% had no migration background. At baseline, 55% (*n* = 107) of participants were aged 2 to 4 years, and 45% (*n* = 87) were aged 5 to 6 years, with 51% (*n* = 99) being female. At the 1-year follow-up, 17% (*n* = 26) of participants were aged 2 to 4 years, and 83% (*n* = 125) were aged 5 to 6 years, with 53% (*n* = 80) being female. At baseline, 92% of participants were classified as having normal weight, 5% were classified as overweight/obese, and 3% had missing BMI data. The prevalence of normal weight and obesity remained relatively stable at 93% and 5%, respectively, with 2% of children having missing BMI data at follow-up.

#### Reporting of PA, sleep and mental health outcomes

Descriptive results for PA, SED, and sleep as well as SDQ scores are reported in Table 2. All PA metrics were moderately higher at baseline compared to FUp, with the sleep metrics remaining unchanged between the two timepoints. At baseline, the study population spent on average 418.7 min/day inactive, 112.7 min/day in LPA, 252.6 min/day in MVPA, and 8.0 h/day asleep, with a SE of 80%. At FUp, the children spent on average 408 min/day inactive, 154.5 min/day in LPA, and 264.5 min/day in MVPA, and showed similar sleep characteristics. Average SDQ scores slightly decreased at FUp from 8.0 to 7.0.

#### PA predicting sleep, SED, and mental health outcomes

Table 3 presents associations of selected covariates on all four outcomes.

**Table 3:** Results of the linear mixed models considering associations of covariates with sleep, SED, and SDQ

Covariate	SDQ			SE		
	Estimate	95% CI	*p*-value	Estimate	95% CI	*p*-value
Sex; female (ref: male)	−1.72	(−2.83; −0.61)	0.003	0.12	(−0.0001; 0.024)	0.052
Age; 5/6 year (ref: 2–4 years old)	−0.59	(−1.37; 0.20)	0.14	0.004	(−0.0056; 0.014)	0.39
BMI; overweight / obese (ref: normal weight / thinness)	1.07	(−0.92; 3.07)	0.29	0.015	(−0.010; 0.041)	0.23
Urbanity; city (ref: rural)	0.60	(−0.56; 1.76)	0.31	−0.012	(−0.024; 0.001)	0.071
*Parental education (ref: medium)*						
High	−0.72	(−2.08; 0.65)	0.30	−0.005	(−0.020; 0.010)	0.49
Low	2.11	(0.003; 4.22)	0.049	−0.002	(−0,025; 0.021)	0.86
Missing	0.73	(−1.98; 3.44)	0.60	0.006	(−0.027; 0.039)	0.72
*Income (ref: medium)*						
High	0.46	(−0.098; 1.91)	0.53	−0.004	(−0.019; 0.012)	0.65
Low	3.35	(1.61; 5.08)	0.0002	−0.013	(−0.032; 0.0060)	0.18
Missing	0.65	(−1.61; 2.90)	0.57	−0.031	(−0.055; -0.006)	0.015
*Migration background (ref: one or two-sided)*						
No migration background	0.24	(−1.24; 1.72)	0.75	−0.0116	(−0.028; 0.0049)	0.17
Missing	2.34	(−0.35; 5.03)	0.087	−0.0055	(−0.037; 0.0263)	0.74

Covariate	TST			SED		
	Estimate	95% CI	*p*-value	Estimate	95% CI	*p*-value
Sex; female (ref: male)	0.18	(0.0169; 0.345)	0.031	4.37	(−17.7; 26.4)	0.696
Age; 5/6 year (ref: 2–4 years old)	0.026	(−0.103; 0.155)	0.69	−20.2	(−40.3; −0.08)	0.049
BMI; overweight / obese (ref: normal weight / thinness)	−0.167	(-0.503; 0.169)	0.33	12.45	(−33.9; 58.8)	0.598
Urbanity; city (ref: rural)	0.024	(−0.146; 0.193)	0.78	−1.02	(−24.0; 21.9)	0.93
*Parental education (ref: medium)*						
High	−0.013	(−0.188; 0.214)	0.90	−13.4	(−40.5; 13.7)	0.33
Low	0.119	(−0.198; 0.436)	0.46	−19.5	(−61.9; 22.9)	0.37
Missing	0.080	(−0.370; 0.531)	0.73	−24.2	(−80.0; 31.7)	0.395
*Income (ref: medium)*						
High	−0.065	(−0.149; 0.279)	0.55	2.79	(−25.7; 31.3)	0.85
Low	−0.085	(−0.344; 0.175)	0.52	18.2	(−16.9; 53.2)	0.31
Missing	−0.052	(−0.386; 0.283)	0.76	6.56	(−38.2; 51.3)	0.77
*Migration background (ref: one or two-sided)*						
No migration background	−0.0326	(−0.257; 0.192)	0.78	−13.8	(−43.3; 15.7)	0.36
Missing	0.0998	(−0.330; 0.530)	0.65	31.1	(−23.6; 85.6)	0.26

SED, sedentary behaviour; SE, sleep efficiency; TST, total sleep time; SDQ , Strengths and Difficulties Questionnaire; Effect; fixed effect estimate; CI; confidence interval

Girls showed lower SDQ, but children of parents with low educational background or low income had higher SDQ.

Results from the LMM analysis are reported in Table 4. We observed no associations of LPA, MVPA, and TPA with SDQ scores. For sleep outcomes (SE or TST) as well as for SED, we found positive associations particularly for MVPA, and TPA with SE and TST and for all PA variables with SED, which were consistent between crude models and adjusted models. (see Table 4 for details).

**Table 4:** Results of the linear mixed model considering crude and adjusted; physical activity intensities predicting sleep, SED, and mental health outcomes

Predictor	Response	Crude effect estimates (95% CI)	*p*-value	Adjusted effect estimates* (95% CI)	*p*-value
LPA	SDQ	0.0097 (−0.014; 0.033)	0.418	0.0127 (−0.017; 0.032)	0.556
MVPA	SDQ	−0.0037 (−0.0010; 0.0022)	0.217	0.00314 (−0.010; 0.0024)	0.224
TPA	SDQ	0.0032 (-0.0010; 0.0028)	0.265	−0.0038 (−0.0104; 0.00277)	0.256
LPA	SE	−0.00031 (−0.0006; −0.00002)	0.039	−0.00031 (−0.00063; 0.000007)	0.55
MVPA	**SE**	**0.00015** **(0.000008; 0.00022)**	**< 0.0001**	**0.00017** **(0.00010; 0.00025)**	**< 0.0001**
TPA	**SE**	**0.00004** **(0.00008; 0.00023)**	**< 0.0001**	**0.00017** **(0.00009; 0.00026)**	**< 0.0001**
LPA	**TST**	**−0.0098** **(−0.013; −0.0061)**	**< 0.0001**	**−0.0105** **(−0.0145; −0.0065)**	**< 0.0001**
MVPA	**TST**	**0.00045** **(0.0016; 0.00337)**	**< 0.0001**	**0.00248** **(0.00152; 0.00345)**	**< 0.0001**
TPA	**TST**	**0.00217** **(0.0012; 0.00317)**	**< 0.0001**	**0.00210** **(0.0010; 0.00317)**	**0.0002**
LPA	**SED**	**0.278** **(0.422; 1.52)**	**0.0005**	**1.014** **(0.430; 1.60)**	**0.0007**
MVPA	**SED**	**−0.741** **(−0.858; −0.623)**	**< 0.0001**	**−0.747** **(−0.871; −0.624)**	**< 0.0001**
TPA	**SED**	**−0.726** **(−0.849; −0.602)**	**< 0.0001**	**−0.743** **(−0.873; −0.612)**	**< 0.0001**

SED; sedentary behaviour; LPA ; light physical activity (PA), MVPA; moderate-vigorous PA; TPA; total PA; SE ; sleep efficiency; TST ; total sleep time; SDQ ; Strengths and Difficulties Questionnaire; Effect; fixed effect estimate; CI ; confidence interval

*All models were adjusted for sex, age, and migration background of child, urbanity, parent education, and monthly household income. Bold = statistically significant, *p* < 0.05

## Discussion

Our study aimed to explore whether device-based measured physical activity was associated with SED, nighttime sleep, and mental health in young German children using repeated measures from a health survey. To our knowledge, this is the first study to investigate the relationship of PA with SED and nighttime sleep as well as mental health in the same population of two- to six-year-old children using device-based measures. Overall our analyses found positive associations between PA and sleep outcomes, showing that children who engaged in high intensities of PA and for longer durations would experience improved nighttime sleep quality and duration.

Current studies examining associations between device-based measured PA and sleep in young children have reported equivocal findings [7]. Duraccio and Jensen, [13] found no association between PA regularity and total nighttime sleep but concluded that sufficient sleep duration was more likely to be achieved for those who spent less time sedentary [13]. In another sample of two- to six-year-olds, no association between PA and sleep duration or efficiency was reported, although sleep latency (the time it takes to fall asleep after turning off the lights) was prolonged when participants engaged in greater amounts of MVPA [14].

In addition, Miller et al., [42] studied three- to six-year-old children and found a favourable association where TPA was positively associated with SE, but not TST. In contrast, Williams et al., [15] found an inverse relationship between the most physically active children and their TST using accelerometry. The authors speculate that PA may promote better sleep quality rather than longer sleep durations; however, these are, at present, untested hypotheses and require further investigation.

Inconsistencies in reported findings throughout the literature may be attributed to variations in accelerometer wear location, device wear time duration, and data processing procedures ([42]; [16]), as well as the considerable variability in both PA and sleep patterns among young children [43, 44] and thus further research is needed to solidify the understanding of this relationship.

PA levels and duration can vary among children due to factors such as parental influences, outdoor climate, the built home environment, and screen time [45–47]. Similarly, differences in nighttime sleep may arise from variations in bedtime routine habits, nutrition, sleeping environments, and individual needs, making it challenging to generalize findings across populations [48].

Exploring the association of PA and mental health, measured by the SDQ, did not show any association. Children’s SDQ scores were within the normal range at both baseline and FUp, with a decrease over time, indicating an average improvement in their emotional and behavioural difficulties. A recent meta-analysis by [49] reported similar results and suggested that this may be due to the relatively small effects of PA on mental health, as younger populations tend to experience relatively high baseline levels of mental health. While previous studies have found positive associations between PA and mental health in older children and adolescents [50, 51], the evidence for this relationship in two- to six-year-olds is scarce [49]. Recent evidence from a preschool population suggests that higher levels of device-based measured MVPA are associated with fewer overall emotional and behavioural difficulties, as measured by the SDQ, with effects becoming more pronounced beyond a threshold of approximately 30 min of MVPA [52].

Although no associations were found, it is plausible that in early childhood, mental health may be more greatly impacted by additional factors, such as the home environment, relationships with others, social interactions, or the various developmental processes occurring in the early years [53], rather than by PA duration and intensities alone. Moreover, it may be that while PA positively influences certain components of mental health (like behavioural regulation), the effects may be nuanced and not captured by broader measures like the SDQ as a measure of mental health in younger children. Although it is a validated tool to determine emotional and behavioural difficulties in children [54], it may not be sensitive enough to detect subtle changes in mental health that could result from fluctuations in PA within the developmental stages of early childhood. Future research should integrate more sensitive and comprehensive mental health measures, such as those assessing behavioural regulation or emotional competence (e.g. Emotion Regulation Questionnaire for Children and Adolescents (ERQ-CA); [55], to provide deeper insights into the subtleties of the PA and mental health relationship in early childhood.

Eventually, ceiling effects may have influenced our findings on the associations of PA, with SED, sleep, and mental health in our pre-school sample. At baseline and FUp, the children in our study were generally active, with most meeting or exceeding the WHO’s recommendation for children of 60 min of daily MVPA [16]. Our population also demonstrated good sleep quality, in general maintained a normal BMI, normal SDQ scores, and came from families with medium to high education levels and high household incomes. Future research should aim to include a more diverse sample, particularly including children who are less active, have poorer sleep quality and quantity, have higher BMI, poorer mental health, or come from socioeconomically disadvantaged backgrounds, to better capture the full spectrum of interactions between PA, SED, sleep, and mental health during the early years.

### Limitations

Limitations should be considered when interpreting our results. First, every effort was made to collect as much data as possible for PA, SED, sleep, and mental health; however, due to stringent inclusion and exclusion criteria, challenges with accelerometer-wear compliance among young children, loss of accelerometers, and considerable daycare facility staff and parental involvement, achieving this was not always feasible at the two timepoints. Our sample population also consisted predominantly of children from high-income German households, which may restrict the generalizability of our findings to broader populations. Additionally, while the SDQ is a validated tool for assessing mental health in children, the version used here has been validated for children aged 4 + years and it relies on parental-reporting rather than clinical diagnosis and thus may introduce potential bias into the results. Further, the statistical analyses in our study were strongly underpowered, since the underlying study was not designed to investigate accelerometer outcomes and only 212 children provided accelerometer data in the subsample. Hence, our results need to be considered in an exploratory manner, but can provide important insights for the design of large-scale studies on device-based measured health behaviours.

Due to missing sleep diaries, we did not account for daytime naps; thus, it is possible that a child’s daily *total* sleep duration within a 24-hour period was not fully captured. Incorporating previously published age-appropriate daytime nap correction factors into participants’ daily TST would help address potential discrepancies not reflected in nighttime sleep data alone. This is a clear limitation of the current study, especially as it is typical for many younger children to nap within a daycare setting [56]. Omission of these sleep periods may have introduced inconsistencies in the associations between PA and sleep or, alternatively, naps may have been misinterpreted as non-wear time by GGIR’s algorithms. While a recent paper [57] showed good agreement with gold standard polysomnography readings (intraclass correlation coefficient = 0.67) we do acknowledge the van Hees (2019) sleep algorithm can underestimate total sleep time and sleep efficiency in young children.

Further, caution is warranted when interpreting inactivity as a proxy for SED in this study, as behaviours may have been misclassified [58]. Future directions should include designing interventions that promote sustained PA while minimizing SED, as well as focus on adapting and refining open-source raw accelerometer data analysis packages, such as GGIR [32], to better understand the relationship between PA, SED, and sleep in relation to children’s health in both cross-sectional and longitudinal data sets.

This manuscript focussed on physical activity behaviors and their influence on sleep parameters first as well as mental health. With regard to mental health a comprehensive analysis of 24-hour movement behaviors considering both PA and sleep based on compositional data analysis (CoDa) might provide further insights regarding this field of research. Within CoDa, the interrelation and time constraint of 24-hour movement behaviors are reflected based on variable transformation and e.g. isotemporal substitution modelling which was used in previous studies to identify that reallocation of SED with any small amount of PA was positively associated with mental health in children, as recently published by Wang et al., [52]. This method provides a paradigm shift from previously considering PA behaviors and SED as independent, although together with sleep duration 24-hour movement behaviours are co-dependent within a fixed time constraint and statistical methods such as CoDa should be considered to avoid a multicollinearity bias or multiple testing by investigating separate models for single behaviors [59].

However, given design considerations of our study, we did not conduct CoDa. Firstly, as stated above sleep duration could potentially be underestimated and sleep diaries were not available for a precise quality control which is why we were not able to consistently cover the 24-hour time frame. Secondly, investigating only the composition PA intensities within the constraint of waking hours or including sleep duration would have led to an imbalanced time constraint. The decision between a linear and CoDa should be cautiously reflected as shown by Tomova et al., [60] where a variable time total, e.g. 24-hour vs. the sum of device-based measured behaviors < 24-hour can lead to substantial differences in the results. Lastly, this study only covers a small selective subset of accelerometry samples and more elaborate analyses comparing CoDa and linear regressions should be conducted on a larger scale.

### Strengths

Eventually, the study had several strengths. It is the first to investigate the associations between device-based measured PA and SED, device-based measured nighttime sleep, and mental health using a validated questionnaire in this age group. Device-based measures of behavioural habits is optimal as it eliminates self-reporting bias, increases accuracy by capturing real-time data without reliance on memory, detects subtle sleep, PA, and SED pattern changes which are sometimes missed in self-reports, and standardizes methods across researchers and studies. However, it should be noted that while accelerometry is a more objective measurement tool, data processing requires researchers to select cut-points that may not have not been validated across the full age range of the child participants [16] and make other design and processing decisions that can alter the resulting device-based measures [58]. Additionally, this research utilized the open-source GGIR package to process raw accelerometer data, classifying it into meaningful measures of PA, inactivity, and sleep. This open-source approach enhances comparability across studies that utilize different accelerometers with varying wear locations, further strengthening this study’s findings.

## Conclusion

Our study revealed associations between device-based measured PA duration and intensities with device-based measured sleep duration/efficiency and SED, but did not find any association of PA with mental health in preschool children. These findings highlight the complexity of health behaviours in early childhood and suggest the need for further and upscaled research, to better understand the interaction and reallocation of 24-hour movement behaviors and their relationship with mental health. Future research is needed to address some of the aforementioned age-specific limitations in device-based studies. Future multi-component interventions, such as those combining PA, SED, and sleep modules should be explored to determine potential synergistic benefits for mental health.

## Data Availability

Data cannot be shared openly due to data protection restrictions, but is accessible upon request under the terms of an agreement with the Leibniz Institute of Prevention Research and Epidemiology - BIPS.

## Declarations

### Clinical trial registration

The original study was registered at the German Clinical Trials Register (DRKS00011065) on 16th of September 2016.
